# Improving Output Power of InGaN Laser Diode Using Asymmetric In_0.15_Ga_0.85_N/In_0.02_Ga_0.98_N Multiple Quantum Wells

**DOI:** 10.3390/mi10120875

**Published:** 2019-12-13

**Authors:** Wenjie Wang, Wuze Xie, Zejia Deng, Mingle Liao

**Affiliations:** 1 Microsystem & Terahertz Research Center, China Academy of Engineering Physics, Chengdu 610200, China; xiewuze@mtrc.ac.cn (W.X.); dengzejia@mtrc.ac.cn (Z.D.); liaomingle@mtrc.ac.cn (M.L.); 2 Institute of Electronic Engineering, China Academy of Engineering Physics, Mianyang 621999, China

**Keywords:** asymmetric multiple quantum wells, barrier thickness, InGaN laser diodes, optical absorption loss, electron leakage current

## Abstract

Herein, the optical field distribution and electrical property improvements of the InGaN laser diode with an emission wavelength around 416 nm are theoretically investigated by adjusting the relative thickness of the first or last barrier layer in the three In_0.15_Ga_0.85_N/In_0.02_Ga_0.98_N quantum wells, which is achieved with the simulation program Crosslight. It was found that the thickness of the first or last InGaN barrier has strong effects on the threshold currents and output powers of the laser diodes. The optimal thickness of the first quantum barrier layer (FQB) and last quantum barrier layer (LQB) were found to be 225 nm and 300 nm, respectively. The thickness of LQB layer predominantly affects the output power compared to that of the FQB layer, and the highest output power achieved 3.87 times that of the reference structure (symmetric quantum well), which is attributed to reduced optical absorption loss as well as the reduced vertical electron leakage current leaking from the quantum wells to the p-type region. Our result proves that an appropriate LQB layer thickness is advantageous for achieving low threshold current and high output power lasers.

## 1. Introduction

InGaN-based multi-quantum well (MQW) laser diodes (LDs) have drawn much attention in recent years due to their potential as the light sources in the applications of high-density optical storage systems, laser printing, full-color displays, small portable projector, among the others [1,2,3,4,5,6,7,8,9,10]. To date, although the InGaN based blue LDs are already commercialized and the material growth technology has been significantly improved, the factors to achieve optimal optoelectronic performance have not been fully developed. There are still many places that need to be improved. In general, high output power and low threshold current are two key indicators to attain superior laser diode performances. The emission mechanism of the InGaN/(In)GaN multiple-quantum-well structure is important and necessary for further improving the performance of LD. The material composition, number, and thickness of the multiple quantum well (WQW) in the active region are crucial structural parameters for optimizing the structure of LD because they directly affect the optical and electrical properties of the device. Among these parameters, the composition fluctuation of the quantum well mainly affects the wavelength of the laser emission, because the band gap of the quantum well varies with composition. Meanwhile, the effect of QW number on the performance of MQW LDs has also been studied both theoretically and experimentally [11,12,13], which indicates that the blue-violet laser (emission wavelength between 392 nm and 420 nm) with two InGaN wells could obtain the lowest threshold current when the band gap ratio is 7/3. But the QW number is highly dependent on other parameters such as laser material, output wavelength, structural design, and so on [14]. When the emission wavelength is around 416 nm, our simulation results and experiments show that the performance of the laser device obtained from the three quantum wells structure is slightly better than that of the two quantum wells structure. Thereby, the InGaN lasers with wavelengths around 416 nm generally employ two or three quantum wells structure [15]. Meanwhile, Alahyarizadeh’s research shows that the thickness of QW can also manipulate the emission characteristic of the InGaN QW [16]. In addition, the experimental data shows that the threshold current is the smallest when the width of the quantum well is around 3 nm, which is consistent with the high-quality sample results [17]. Despite all these findings, the influence of barrier parameters is less studied, especially the thick barrier. The current research mainly focused on improving the carrier distribution by the special composite barrier layer [15], and few on the distribution of the optical field. The main reason is that the common barrier layer is too thin relative to the waveguide layer and the cladding layer, and the effect on the distribution of the optical field is relatively small.

In principle, the barrier layer of MQW structure affects the asymmetric distribution of carrier concentration in the p-doped and n-doped regions and the optical field distribution over the whole LD structure. Due to the huge difference between electron (600 cm^2^/Vs) and hole mobility (10 cm^2^/Vs) [18], the carrier distribution in the MQW region is non-uniform. The concentration of holes in the n-doped region is significantly lower than the concentration of electrons in the p-doped region. The asymmetry of the carrier distribution in the MQW and the optical field distribution deviating from the center of the quantum well are thus enhanced, and the luminous efficiency would be reduced [19]. Since the barrier thicknesses of the conventional InGaN/GaN MQW LDs are usually thin (8~20 nm), it is difficult to achieve effective carrier transport into the MQW near the n-layer. So, the actual carrier density will not be uniform in every QW, and it is hard to affect optical field distribution and the associated absorption loss. Many researchers have improved the performance of LD by inserting an unintentionally doped (In) GaN or AlGaN thin layer between LQB and EBL, or thickening of the upper and lower waveguide layers to reduce optical absorption loss and expand the distribution of the optical field [20,21,22]. However, it is difficult to further reduce optical absorption loss and improve the optical field distribution in these device structures, because the upper waveguide layer is always p-type with a high absorption coefficient. It is more convenient and effective to improve the optical field distribution by adjusting the parameters of the quantum well region with a low absorption coefficient. For the sake of eliminating the unfavorable effect of the asymmetric carrier and optical field distribution mentioned above, the MQW active region needs to be redesigned and optimized.

In this letter, the relative thickness of the first or last barrier layers in the three In_0.15_Ga_0.85_N/In_0.02_Ga_0.98_N quantum wells laser diode, are designed and optimized to enhance the optical field distribution into the MQW while reducing the optical absorption loss. Simultaneously, by redesigning the barrier layer, the comparison between their optical and electrical characteristics (especially leakage current) are analyzed and compared with the theoretical simulation results.

## 2. Device Structure and Simulation Setup

**Figure 1:**
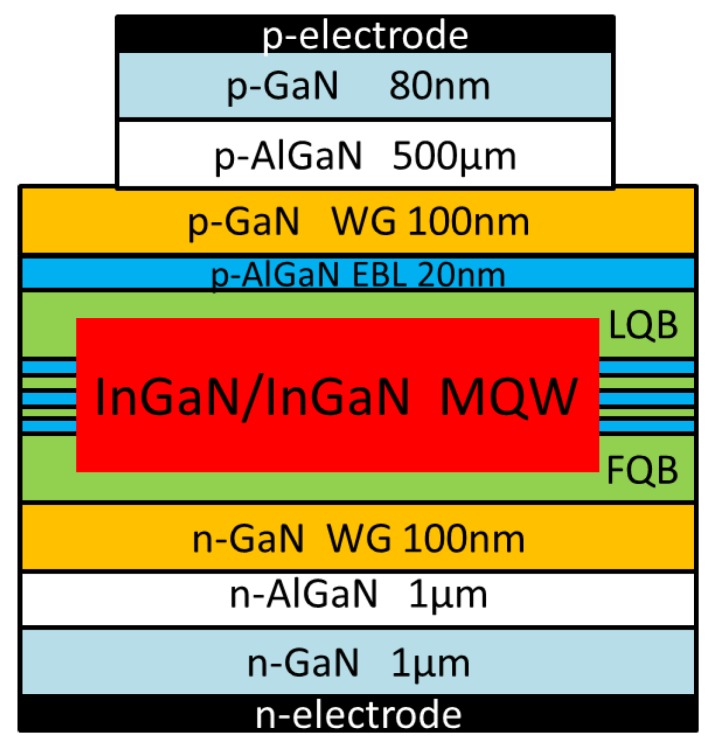
Schematic of In_0.15_Ga_0.85_N/In_0.02_Ga_0.98_N quantum well laser diodes.

For the illustration purpose, a structure of three In_0.15_Ga_0.85_N/In_0.02_Ga_0.98_N quantum wells laser diodes with various first or last barrier layers are designed to increase the optical field distribution in the active region, and to reduce the optical absorption loss and the vertical electron leakage current. For comparison with symmetric quantum well structure, there are series of structures with various first or last barrier layers of In_0.15_Ga_0.85_N/In_0.02_Ga_0.98_N quantum wells laser diodes shown in Figure 1. These laser structures with varying thicknesses of quantum barrier layers consist of a GaN free-standing substrate, a 1 μm thick GaN buffer layer, an n-Al_0.08_Ga_0.92_N confinement layer with a thickness of 1 μm and the doping concentration of 3 × 10^18^ cm^−3^, an n-type GaN lower waveguide (LWG) layer with a thickness of 100 nm and the doping concentration of 1 × 10^18^ cm^−3^, a three-period 3 nm In_0.15_Ga_0.85_N well/15 nm In_0.02_Ga_0.98_N barrier quantum well with background concentration of 5 × 10^16^ cm^−3^, a p-Al_0.2_Ga_0.8_N electron blocking layer (EBL) with 20 nm thickness, a p- GaN upper WG (UWG) layer with 100 nm thickness, a p-AlGaN confinement layer with 500 nm thickness, and a p-GaN contact layer with 80 nm thickness. The doping concentration of all p-type layers is 5 × 10^18^ cm^−3^.

The differences in these laser structures are only the first or last barrier thickness of the In_0.15_Ga_0.85_N/In_0.02_Ga_0.98_N quantum wells. In the reference structure (marked as FQB-15 or LQB-15), the active region of MQW is symmetrical and the barrier layer thicknesses is 15 nm. In the first barrier series of the LD structures (marked as FQB-XX) and the last barrier series of the LD structures (marked as LQB-XX), the first or last barrier is an In_0.02_Ga_0.98_N single layer with different thickness.

Asymmetric In_0.15_Ga_0.85_N/In_0.02_Ga_0.98_N MQW samples with various barrier layer thicknesses, photoelectric properties of these laser structures, including the optical filed distributions, optical confinement factors, peak modal gains, electron leakage currents and output powers, are theoretically simulated by a powerful semiconductor laser simulation tool Crosslight Device Simulation Software (Crosslight Software Inc., Vancouver, Canada) [23,24]. In order to eliminate the influence of the contact, both the p and n electrodes are set as an ideal Ohmic contact type. The cavity length and ridge width are set to be 800 μm and 2 μm, respectively. The reflectivity of both sides of the laser cavity is set to 19%. The screening factor is set to be 0.25 [25], and the band offset ratio (ΔE_c_/ΔE_g_) is set to be 0.67 [26]. Meanwhile, for the n-type and p-type layers, their absorption coefficients are set as 5 cm^−1^ and 50 cm^−1^ [27], respectively. The absorption coefficient of highly doped EBL is 100 cm^−1^.

## 3. Results and Discussions

**Figure 2:**
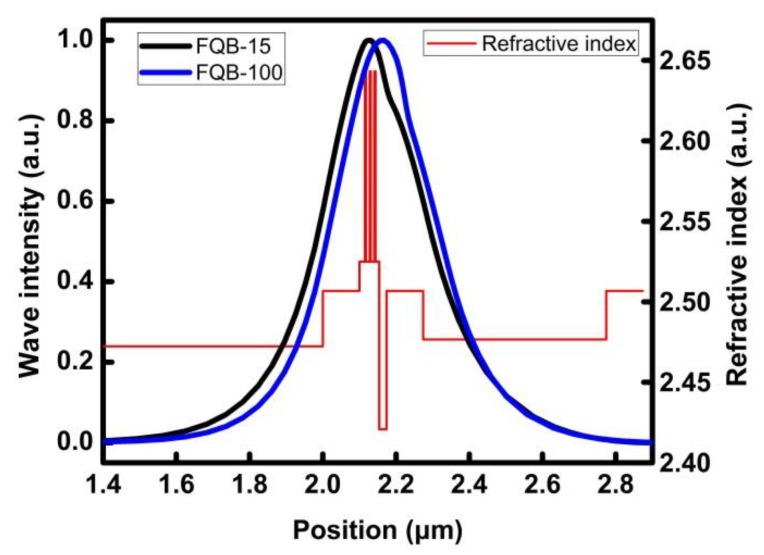
The refractive index profile of the symmetric quantum well LD structure (red line) and the optical field distributions of the FQB-15 and FQB-100 structures.

First, the effect of the layer thickness of the first quantum barrier layer is investigated. The optical characteristics of the four FQB thicknesses of 15 nm, 45 nm, 100 nm and 225 nm are simulated, respectively. Then, three In_0.15_Ga_0.85_N/In_0.02_Ga_0.98_N quantum wells with LQB thicknesses of 100 nm, 300 nm and 500 nm, respectively, are calculated. The refractive index profile of the symmetric quantum well LD structure (red line) and the optical field distributions of the FQB-15 and FQB-100 structures are shown in Figure 2. The refractive indices of InN, GaN and AlN are set as 3.4167, 2.5067, and 2.0767, respectively. The refractive indices of In_x_Ga_1-x_N and Al_x_Ga_1-x_N are calculated using an approximate method as follows:

(1) $$n\left({\mathrm{In}}_{x}{\mathrm{Ga}}_{1-x}N\right)=\left[n\left(\mathrm{InN}\right)-n\left(\mathrm{GaN}\right)\right]\cdot x+n\left(\mathrm{GaN}\right)$$

(2) $$n\left({\mathrm{Al}}_{x}{\mathrm{Ga}}_{1-x}N\right)=\left[n\left(\mathrm{AlN}\right)-n\left(\mathrm{GaN}\right)\right]\cdot x+n\left(\mathrm{GaN}\right)$$

According Equations (1) and (2), the refractive indices of the Al_0.2_Ga_0.8_N, Al_0.07_Ga_0.93_N, Al_0.08_Ga_0.92_N, In_0.15_Ga_0.85_N and In_0.02_Ga_0.98_N are calculated to be 2.4207, 2.4766, 2.4723, 2.6432 and 2.5249, respectively. Due to the large refractive index difference between the GaN waveguide layer and the upper and lower confinement layers, most of the optical field is located to the layer between the upper and lower GaN waveguides. However, the appearance of the low refractive index Al_0.2_Ga_0.8_N EBL decreases the mean value of the p-type refractive index, resulting in an asymmetric optical field distribution on the left and right sides of the quantum wells, and the peak is not situated at the middle of the quantum wells. The peak position shifted slightly to the n-type side, and the optical limit factor (OCF) did not reach the maximum value. In addition, a large part of the optical field is distributed in the p region with a large absorption coefficient, which causes a large optical absorption loss and the laser’s threshold current rises.

When the FQB or LQB layer becomes thicker, the EBL or n-GaN WG will move away from the quantum wells. The optical field peak moves to the p-type region, so that more optical fields are distributed in the quantum well region, and the OCF is enhanced. In addition, since the optical field is close to a Gaussian distribution, when the FQB or LQB layer becomes thicker, the optical field proportion of the p-type region decreases significantly, thereby reducing light absorption loss.

**Figure 3:**
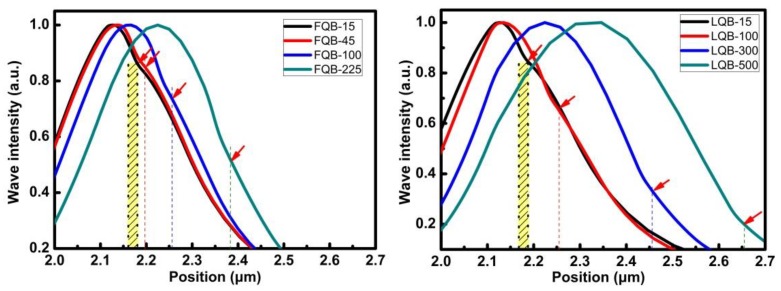
Optical field distribution of FQB or LQB structures (dashed lines and red arrows indicate the position of the electron blocking layer.).

**Figure 4:**
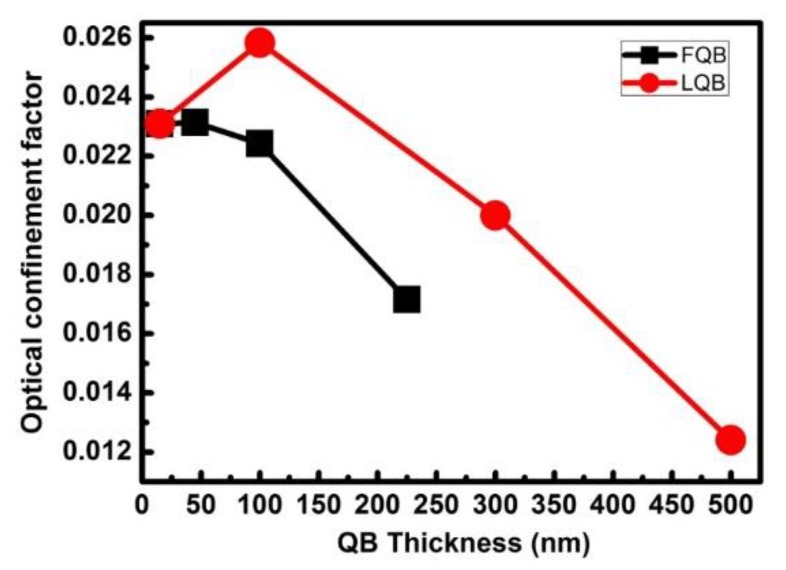
Optical confinement factor of LDs with different FQB or LQB layer thicknesses.

However, the FQB or LQB layer should not be too thick, either. As the thickness of the FQB or LQB layer increases, the optical field is further deviated from the quantum wells. Meanwhile, the dotted line in Figure 3 indicates that the peak position of the light field will pass through the quantum wells region and continue to move to the p region, moving to the position between the quantum wells and EBL. It can be seen that with the increase of the FQB or LQB layer thickness, the optical field is more distributed inside the FQB or LQB layer. However, the optical field peak has deviated from the quantum wells. Even if more optical fields are distributed inside the FQB or LQB layer, the optical absorption loss further decreases, but the OCF may be reduced by the shift of the peak position of the optical field [28]. This is consistent with the trend of OFC in Figure 4 as the thickness of the barrier layer changes. Therefore, with the increase of the FQB or LQB layer thickness, the peak position of the optical field moves from the n-type regions to the p-type regions, and the optical confinement factor slowly grows to the maximum and then decreases rapidly.

**Figure 5:**
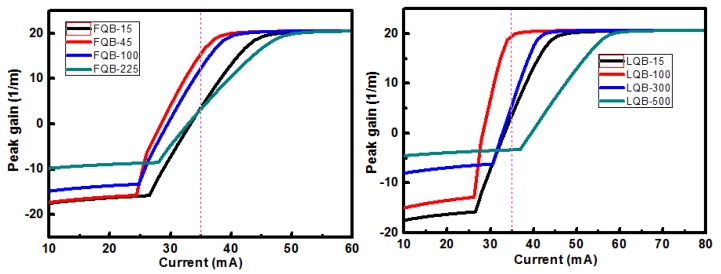
Peak modal gain of LDs with different FQB or LQB layer thicknesses.

The peak modal gain of LDs with different FQB or LQB layer thicknesses are shown in Figure 5. When the optical confinement effect in the quantum well region is enhanced, the modal gain will increase accordingly, because the modal gain is the product of OCF Γ_0_ and the material gain g of the quantum well. Taking a specific injection current value of 35 mA as an example, the calculated peak modal gain of four lasers with varying FQB or LQB layer thicknesses are compared, as shown by the red dotted line in Figure 5. When these structures are injected with the same current, the peak modal gain grows from 4 to 20 m^−1^ and the corresponding LQB layer thickness increases to 100 nm. When the thickness of the LQB layer continues to increase to 500 nm, the peak modal gain decreases from 20 to −3 m^−1^. In addition, when the thickness of the FQB layer exceeds 45 nm, the peak modal gain will decrease, which confirms that the optical field shift will not be beneficial to OCF. This is consistent with the trend of the OFC in Figure 4.

**Figure 6:**
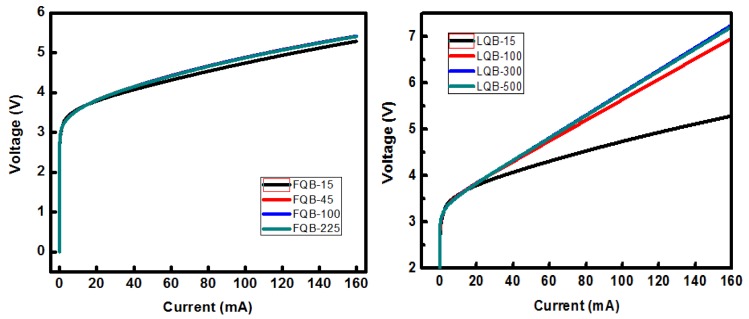
V-I characteristic of lasers with varying FQB and LQB thickness.

**Figure 7:**
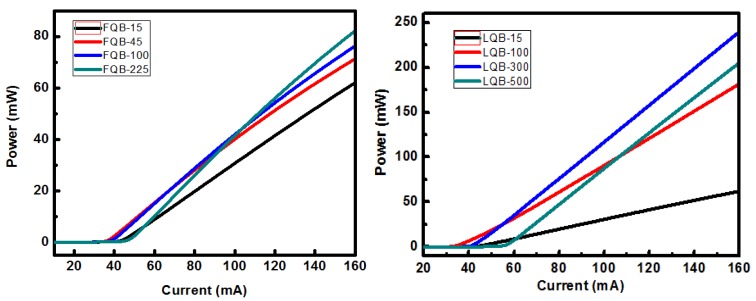
L-I characteristics of lasers with varying FQB or LQB layer thicknesses.

The changes in OCF and optical absorption loss (OAL) have a large effect on the electrical characteristics of the In_0.15_Ga_0.85_N/In_0.02_Ga_0.98_N QW LD. Figure 6 shows a completely different V-I characteristic of lasers with varying FQB and LQB thickness. In the laser structure of this paper, the quantum wells region is close to the electron blocking layer. Increasing the thickness of LQB makes the distance of the electrons reflected by the EBL to the well layer larger, resulting in greater resistance. However, increasing the thickness of FQB has little effect on the reflection of EBL on electrons, and the resistance is basically unchanged. This is quite different from the electron-overflow-suppression (EOS) layer [22] above the upper waveguide layer. Reference [22] indicates that the EOS layer can eliminate the voltage rise only when the EOS layer is located between the upper waveguide layer and the p-doped region. L-I characteristics of lasers with varying FQB or LQB layer thicknesses are shown in Figure 7. When the modal gain and loss are completely balanced, the modal gain tends to saturate and the laser reaches the threshold condition [29]:

(3) $$\Gamma_{0}g=\alpha_{i}+\alpha_{m}$$

where $\alpha_{i}$ is the optical absorption loss, $\alpha_{m}$ is the mirror loss. As the thickness of the FQB and LQB layers increases, the threshold current will increase first and then decrease. The threshold currents of lasers with FQB thicknesses of 15, 45, 100, and 225 nm are 48, 39, 42, and 50 mA, respectively. Similar trends also occur when the thickness of the LQB changes. When the thickness of LQB is 100, 300, and 500 nm, the corresponding threshold currents are 36 mA, 42 mA, and 57 mA, respectively. In particular, LQB-300 has a lower threshold current density than the reference structure when the OFC value is smaller than that of the reference structure, due a small $\alpha_{i}$. The change trend of the laser output power in Figure 7 is similar to the threshold current trend in Figure 5. When the thickness of LQB is 300 nm, the LD device achieves the highest output power of 240 mW.

**Figure 8:**
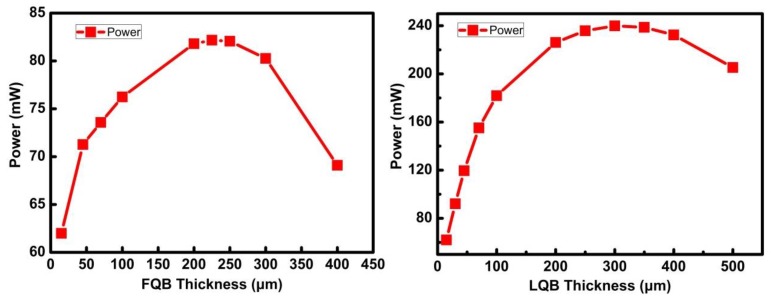
Variation in L-I characteristics with different FQB or LQB layer laser structure under an injection current of 160 mA.

The optimum thickness value of the FQB or LQB layer can be determined based on the changes in the OAL and OCF. The L-I characteristic curves of asymmetric quantum well LDs with FQB or LQB layers of various thicknesses are calculated. Figure 8 depicts the variation in L-I characteristics with different FQB or LQB layer laser structure under an injection current of 160 mA. It can be seen that the laser output power increase evidently as the thickness of FQB (or LQB) layer increases from 15 nm to 225 nm (or 300 nm). That is, when the FQB (or LQB) layer gradually increases from the initial thickness of 15 nm, it will bring two factors that increase the optical confinement factor and decrease the absorption loss, which are conducive to increasing the output power. For the asymmetric quantum well LDs with an FQB (or LQB) layer thicker than 300 nm (or 400 nm), the deviation of the peak position of the optical field from the quantum wells caused a sharp decrease in OCF, which led to a rapid decrease in the output power of the laser. There is a relatively stable output power area between the two regions where the output power changes rapidly, that is, 200 nm to 250 nm for the LBQ layer and 250 nm to 350 nm for the FBQ layer. This is the result of the comprehensive effects of the disadvantage of OCF reduction and the positive compensation of optical absorption loss decrease. When the thickness of the FQB or LQB layer is further increased, the output power drops, which is caused by the rapid decline of OCF is not enough to be compensated by the reduction of the optical absorption loss, so the output power of the asymmetric quantum well laser decreases. The optimum thickness of the FQB (or LQB) layer in the laser structure is around 225 nm (or 300 nm). However, the thickness of the LQB layer is more important than the FQB layer. At 160 mA, the maximum output power of the LQB-300 is 240 mW, which is 2.92 times that of the FQB-225 layer, and 3.87 times that of the reference structure.

**Figure 9:**
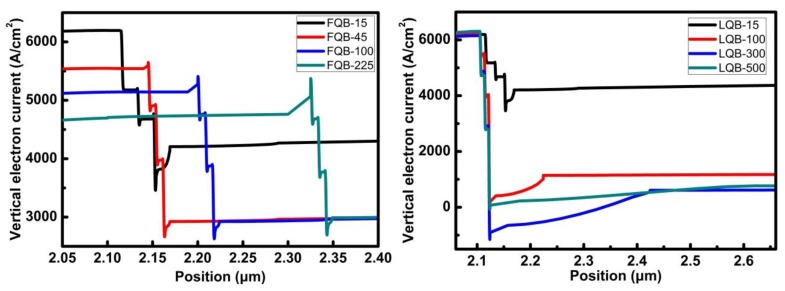
Vertical electron current density of LDs with different FQB or LQB layer thickness.

In order to compare the impact of the first and last barrier layer thickness on the output power, the vertical electron current density of the different FQB and LQB layer structures are simulated, as indicated in Figure 9. It indicates that in the normal working state of the laser, electrons enter the quantum wells from the n-type region, resulting in radiative and non-radiative recombination of electrons and holes in each quantum well, so that the electron current density gradually decreases in the quantum wells. It is noted that the Al_0.2_Ga_0.8_N EBL can effectively confine electrons in the quantum wells, and at the same time, it can reflect electrons back into the quantum wells and recombine with holes, thereby reducing the electron leakage current. The percentage of electron leakage can be calculated by dividing the electron current entering the p-type region from the quantum wells by the electron current injected into the quantum wells. For the 15, 45, 100, and 225 nm FQB layers, the percentages of electron leakage are 67.9%, 52.7%, 56.9%, and 62.8%, respectively. In a series of laser structures with varying FQB thicknesses, when the thickness of the FQB layer exceeds 45 nm, the decrease in electron current density from the bottom quantum well to the top quantum well becomes greater, which indicates that the degree of recombination of electrons and holes is inconsistent in each quantum well. In addition, the blocking ability of Al_0.2_Ga_0_._8_N EBL becomes weaker as the FQB thickens.

**Figure 10:**
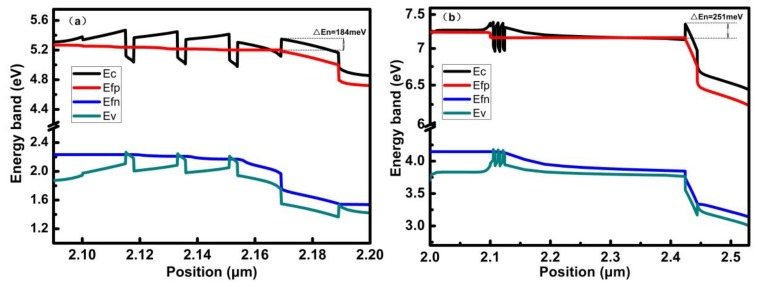
Energy band diagrams of reference LD structure (**a**) and LQB-300 structure (**b**) under an injection current of 160 mA.

The electron current densities of LQB structures are significantly different from that of FQB structures. For the 15, 100, 300, and 500 nm FQB layers, the percentages of electron leakage in the LQB layer changed structure are 67.9%, 18.3%, 9.9%, and 12.1%, respectively. It can be clearly seen that compared with the FQB structure, the electron current density of the LQB structure decreases more, and the change of the electron current density is more obvious in the upper quantum well, indicating that more carriers are recombined in the LQB structure than that of reference structure. This is consistent with the results of the energy band diagrams in Figure 10. Now, the largest effective EBL barrier for electrons in the LQB series is ΔEn = 215 meV in LQB-300, which is much greater than that of the reference LD (ΔEn = 184 meV). A large increase in the effective EBL barrier for electrons results in a significant reduction in electron leakage and improves device performance. The electrons can be effectively reflected to the quantum wells region to increase the effective recombination probability of the two quantum wells near the n-type region, so that the electron concentration distribution in the quantum wells region is more even. This is more advantageous to carrier recombination and device efficiency. When the thickness of the barrier layer is increased to more than 300 nm, the electron leakage current remains substantially unchanged or even slightly increased. It means that, the increase in the thickness of the barrier layer does not effectively reduce the electron leakage, but increases the resistance of the device, so that the threshold current becomes larger, which is consistent with Figure 7. The LQB-300 structure has the smallest electron leakage current in the p region, which indicates that greatly reduced electron leakage is critical to improve the output power of InGaN-based LDs. Therefore, by decreasing optical absorption loss and greatly reducing the electron leakage, combining these two factors, we obtain the optimal structure LQB-300 which produces the maximum power of 3.87 times that of the reference structure power.

## 4. Conclusions

A series of In_0.15_Ga_0.85_N/In_0.02_Ga_0.98_N MQW laser diodes (LDs) with different barrier layers are investigated with the simulator Crosslight. It is found that when the first (or last) In_0.15_Ga_0.85_N barrier layer is no more than 45 nm (or 100 nm), due to the increase in the thickness of the first (or last) barrier layer, the optical field is limited better in the MQW, and the optical absorption loss is reduced. Subsequently, a low threshold current and high output power are achieved. As the thickness of the barrier layer becomes larger, the output powers of the lasers gradually increase, and the positive effects of the reduced optical absorption loss are partially compensated by the negative effects of the OCF reduction. However, when the FQB (or LQB) layer is thicker than 225 nm (or 300 nm), the photoelectric performance of LDs become worse. It is due to the rapid decrement of the OCF, which is not enough to be compensated by the reduction of the optical absorption loss. Nevertheless, compared to the FQB structure, the thick LQB layer will significantly reduce the vertical electron leakage current leaking from quantum wells to p-type region, especially the LQB-300 structure. As a result, the maximum output power of the LQB-300 is 3.87 times that of the reference structure.
